# Internalization and Colocalization of a Polyclonal Antibody Against *Porphyromonas gingivalis* FimA type I in Infected Cells

**DOI:** 10.1055/s-0044-1801302

**Published:** 2025-03-12

**Authors:** Napaporn Apiratmateekul, Kusuma Jamdee, Chantarapim Pinnork, Nawarat Khumyat, Niratcha Chaisomboon, Jantipa Jobsri

**Affiliations:** 1 Department of Medical Technology, Faculty of Allied Health Sciences, Naresuan University, Phitsanulok, Thailand; 2 Reference Material and Innovation Research Unit, Faculty of Allied Health Sciences, Naresuan University, Phitsanulok, Thailand; 3 Research Laboratory, Faculty of Dentistry, Naresuan University, Phitsanulok, Thailand; 4 Department of Oral Biology, Faculty of Dentistry, Naresuan University, Phitsanulok, Thailand

**Keywords:** intracellular bacteria, immunoglobulin, *Porphyromonas gingivalis*

## Abstract

**Objective:**

The aim of this work was to investigate the effect of a rabbit polyclonal antibody specific to *Porphyromonas gingivalis* FimA type I (FimI) protein internalized into *P. gingivalis* infected cells.

**Materials and Methods:**

Rabbits were immunized with *P. gingivalis* FimI protein and the serum was collected for immunoglobulin (Ig) purification. For visualization of the antibody inside the cells, it was labeled with Cy3 dye. Live *P. gingivalis* was labeled with PKH67 dye. Rabbit anti-FimI Ig-Cy3 was internalized into H357 cells infected with *P. gingivalis* -PKH67 by electroporation or coincubation. Location of the Ig or *P. gingivalis* was observed under fluorescence microscope or confocal microscope. Percentage of *P. gingivalis* -PKH67 infected cells was analyzed by flow cytometry.

**Statistical Analysis:**

Normality of data distribution was tested by Shapiro–Wilk test. The data failed normality test and were further analyzed by Kolmogorov–Smirnov test.

**Results:**

Rabbit anti- *P. gingivalis* FimI Ig-Cy3 and *P. gingivalis* -PKH67 were both located next to the nucleus. The rabbit anti-FimI Ig-Cy3 was able to enter H357 cells after the cells were cultured in the medium containing the labeled Ig for 16 hours. The location of the Ig was near the nucleus as found in cells electroporated with the Ig-Cy3. The percentage of *P. gingivalis* -PKH67 infected cells seemed to be decreased after the infected cells internalized anti-FimI Ig by electroporation. However, it was not statistically significance.

**Conclusion:**

Rabbit anti- *P. gingivalis* FimI Ig and *P. gingivalis* was colocalized near the nucleus. And the rabbit anti-FimI Ig was able to enter H357 cells by coincubation method.

## Introduction

*Porphyromonas gingivalis* is a key stone pathogen of periodontitis. 1 *P. gingivalis* is an anaerobic bacterium that expresses many virulent factors including fimbriae. *P. gingivalis* fimbriae plays an important role in adherence to host oral cells, oral tissues, and early bacterial colonizers, thus the bacterium can attach and colonize in the oral cavity. *P. gingivalis* fimbriae are composed of major (FimA) and minor fimbriae, encoded by the fimA gene and the mfa1 gene, respectively. 2 3 4 According to the genotype of FimA, *P. gingivalis* can be divided into six classes, FimA genotype I to V and Ib. 5 FimA genotype II and IV are associated with periodontitis, while type I is found in healthy gingiva. 5 6

After *P. gingivalis* use its fimbriae to adhere and colonize in the subgingival area, where the oxygen is depleted and contains amino acids and hemin in the gingival crevicular fluid, they proliferate and spread. 1 7 8 *P. gingivalis* is able to tolerate and escape from host immune, and induce chronic inflammation of the periodontal tissue. 1 8 9 10 11

Planktonic or extracellular *P. gingivalis* growth can be inhibited by host immune system and antimicrobial substances. 12 13 However, *P. gingivalis* is able to stay alive and proliferate inside many types of host cells including epithelial cells, periodontal ligament cells, and endothelial cells. Since it hides inside the host cell, host immune system and extracellular antimicrobial substances cannot eliminate the bacteria. Thus, host cells become a source of bacterial proliferation. 11 14 15 This causes recurrence of periodontitis after the periodontitis treatment.

In this study, we attempted to determine the effect of antibody specific to *P. gingivalis* FimA on intracellular *P. gingivalis* . Inside the cells, tripartite motif containing-21 (TRIM21) binds to immunoglobulin (Ig) Fc and induces degradation of the protein that is captured by the Ig via proteasome activation. 16 17 18 This mechanism was proposed to explain the ability of cells to eliminate intracellular virus that was captured by the specific antibody. The internalized antibody might also be able to inhibit or kill the intracellular bacteria.

## Materials and Methods

### *P. gingivalis* FimA Type I Immunization

Rabbit immunization with FimA Type I (FimI) was approved by the Naresuan University Institutional Animal Care and Use Committee (project no. NU-AE600814). Histidine-tagged FimI (His-FimI) was amplified from pcDNA3.FimA-PVXCP 19 and inserted into the pET28a plasmid. The recombinant plasmid was purified and transformed into BL21DE3 *Escherichia coli* for His-FimI protein expression. His-FimI was purified using nickel column (Thermo Scientific, United States) and histidine tag was removed by enterokinase enzyme (Bio Basic Inc, Canada). FimI protein was administered intravenously to two female New Zealand white rabbits (Nomura Siam International Co., Ltd., Thailand). FimI protein was diluted to 200 μg in 0.5 mL sterile saline solution and then administered into the marginal ear vein. FimI boosting was done on day 14, 28, and 42 with 100 μg FimI protein diluted in 0.5 mL sterile saline solution. Rabbit blood samples (1 mL) were collected from the marginal ear vein at day 0 as preimmune sera. And at day 49, seven days after the last boost, the rabbit blood samples were collected for antibody titer evaluation. Antibody titer was evaluated by enzyme-linked immunosorbent assay (ELISA). Comparing to preimmune serum, the antibody titer was relatively high. Therefore, the blood could be collected for further Ig purification. The final blood collection and euthanasia were performed on day 55. The serum was prepared by allowing the blood samples to clot at room temperature for 1 hour and spinning at 2,500 revolutions per minute for 15 minutes at room temperature. The serum was transferred to new tubes and kept at –20°C until use.

### Rabbit Anti-FimI Ig Purification

Rabbit anti-FimI Ig was purified from rabbit serum using Protein A Sepharose column (Abcam, United States) according to the manufacturer's instruction. Briefly, the serum was mixed with phosphate buffer saline (PBS) and passed through the column. The column was washed with PBS containing 0.5 M NaCl and the rabbit Ig was eluted from the column using 0.1 M citric acid, pH 2.75. The eluent was neutralized by adding 0.1 volume of 1 M Tris, pH 9.0. The rabbit Ig was concentrated and buffer exchanged to PBS using Pall Macrosep Advance Centrifugal devices with Omega Membrane—10 kDa cutoff (Tisch Scientific, United States). Rabbit anti-FimI Ig was aliquoted and kept at –20°C.

### Enzyme-Linked Immunosorbent Assay with *P. gingivalis*

*P. gingivalis* ATCC 33277 and ATCC 53978 (W50) strains were grown anaerobically at 37°C in a Thermo Scientific anaerobic chamber (Thermo Scientific). Culture media was tryptic soy broth (Becton, Dickinson and Company, United States) supplemented with 5.0 mg/mL of yeast extract (Becton, Dickinson and Company), 0.5 mg/mL of L-cysteine hydrochloride (HiMedia Laboratories Pvt. Ltd, India), 1.0 μg/mL of vitamin K1 (Sigma Chemical Co., St. Louis, Missouri, United States), and 5 μg/mL of hemin (Sigma-Aldrich, United States).

ELISA was performed using formalin-fixed *P. gingivalis* . The bacteria were grown as described above and harvested by centrifugation. The cell pellet was washed once with PBS and the washed cells were incubated with 0.5% formalin in 0.9% NaCl overnight. The formalin-fixed cells were centrifuged and resuspended at 10 ^10^ cells/mL in PBS before use.

For ELISA, 100 μL of formalin-fixed *P. gingivalis* was aliquoted into each well of 96-well ELISA plate and the plate was incubated overnight at 4°C. After washing in PBST buffer (PBS with 0.05% (v/v) Tween-20), the plate was blocked with 2% (w/v) bovine serum albumin in PBS for 1 hour. After washing, 100 μL of rabbit anti-FimI Ig (1 μg/mL) in PBST buffer was added and the plate was incubated at 37°C for 1 hour. After washing, the bound Ig was detected by incubation with peroxidase-conjugated anti-rabbit Ig (Cell Signaling Technology, Massachusetts, United States) at 37°C for 1 hour. After washing, tetramethylbenzidine substrate was added and incubated in dark for 30 minutes. Note that 1 N HCl was added and the OD450 was recorded using a microplate reader.

### *P. gingivalis* Labeling with PKH67 Green Fluorescent

Live *P. gingivalis* was labeled with PKH67 Green Fluorescent (Sigma-Aldrich) following the method provided by the manufacturer with some modifications. One hundred microliters of PKH67 solution were mixed with 1 × 10 ^9^ *P. gingivalis* cells and incubated for 15 minutes at room temperature with protection from the light. The labeling reaction was stopped by addition of fetal bovine serum (FBS). The labeled bacteria were collected by centrifugation and resuspended in H357 cell growth media for further use for invasion into H357 cells.

### Invasion of Labeled *P. Gingivalis* into H357 Cells

H357 is a human tongue squamous cell carcinoma that has been used as a host cell model in *P. gingivalis* host-cell invasion assays. 20 H357 cells were cultured in 1:1 mixture of DMEM (Dulbecco's Modified Eagle Medium) and Ham's F12 media (Gibco, Thermo Fisher Scientific) supplemented with 2 mM glutamine (Gibco), 10% FBS (Hyclone Laboratories Inc, United States), and 0.5 μg/mL sodium hydrocortisone succinate (Sigma-Aldrich). The labeled bacterial suspension then replaced the medium of H357 cells (1.4 × 10 ^6^ cells) that were previously cultured for 24 hours in 60 mm culture dish. H357 cells were incubated with the labeled bacteria for 4 hours before the culture medium containing free bacteria was removed and replaced by complete culture media. Cells were cultured for a period of time desired for each experiment.

### Rabbit Anti-FimI Ig Labeling with Cy3

Rabbit anti-FimI Ig was linked to Cy3 using ReadiLink Rapid Cy3 Antibody Labeling Kit (AAT Bioquest, United States). Fifty microliters of 1 mg/mL of rabbit anti-FimI Ig was mixed with 5 μL of reaction buffer. The mixture was added into a vial containing Cy3 dye and mixed by pipetting. The conjugation reaction mixture was incubated at room temperature for 1 hour. The conjugation reaction was stopped by addition of 5 μL of TQ-Dyed Quench Buffer. The reaction was incubated at room temperature for 10 minutes and the labeling Ig was stored at –20°C.

### Internalization of Rabbit Anti-FimI Ig into H357 Cells by Electroporation

After H357 cells were invaded by PKH67-labeled *P. gingivalis* for 16 hours, cells were harvested by trypsinization and 1 × 10 ^6^ cells were resuspended in 200 μL of Opti-Mem (Gibco). The cell suspension was mixed with 8 μL of 0.5 mg/mL of rabbit anti-FimI Ig or rabbit anti-FimI Ig-Cy3. Cells were electroporated with square wave pulse type at 160 V for 15 ms using Gene Pulser Xcell (Bio-Rad Laboratories, Inc., United States). After electroporation, 0.5 mL of media was added and the cells were transferred to a 35-mm culture dish or 8-well Laboratory-Tek II Chamber Slide (Nunc, United States).

### Cell Visualization Under Microscope

For observing *P. gingivalis* -PKH67 and anti-FimI Ig-Cy3 inside the cells, cells were visualized using Zeiss Observer.D1 AXIO with Zen2 Software (Zeiss, Germany). In addition, cells containing *P. gingivalis* -PKH67 and anti-FimI Ig-Cy3 cultured in 8-well Laboratory-Tek II Chamber Slide were fixed with 0.36% formaldehyde in PBS for 10 minutes, permeabilized with 0.1% Triton X-100 in PBS for 1 minute, and stained with 4′,6-Diamidino-2-Phenylindole, dihydrochloride (1:1,000 in PBS containing 0.2% Tween-20) for 10 minutes. The slide was mounted with mount solution before visualization with Nikon A1R confocal microscope and NIS-Element AR software (Nikon, Japan).

### Internalization of Cy3-Rabbit Anti-FimI Ig into H357 Cells by Coincubation

After H357 cells were invaded by PKH67-labeled *P. gingivalis* for 16 hours, culture medium was replaced with culture medium containing 2.5 μg/mL of anti-FimI Ig-Cy3. Cells were cultured for further 16 hours and the medium containing the Ig was removed and replaced with normal culture medium. Cells were observed under Zeiss Observer.D1 AXIO with Zen2 Software.

### Flow Cytometry Analysis of H357 Cells Containing *P. gingivalis*

After H357 cells containing *P. gingivalis* -PKH were electroporated with rabbit anti-FimI Ig, cells were cultured up to 6 days before analysis with BD FACSAria III and FACSDiva 8.0.1 software (BD Biosciences, United States). Cells were trypsinized and resuspended in PBS. Fifty thousand single cells were analyzed and fluorescein isothiocyanate signals were counted as H357 cells containing *P. gingivalis* -PKH.

### Statistical Analysis

Normality of data distribution was tested by Shapiro–Wilk test. The data failed normality test and were further analyzed by Kolmogorov–Smirnov test. All statistical analyses were performed using GraphPad Prism software version 10.2.3.

## Results

### Specificity of Rabbit Anti-FimI Ig

Binding of rabbit anti-FimI Ig to *P. gingivalis* strain ATCC33277, harboring FimA type I, and strain ATCC53978 or W50 strain, harboring FimA type IV, was assessed by ELISA. The antibody binding gave OD450 3.55 ± 0.08 when the antigen tested was ATCC33277 strain. In contrast, the OD was only 1.34 ± 0.10 when the antigen tested was W50 strain. This data showed that rabbit anti-FimI Ig was specific to FimI harboring strain and could bind at a certain level to FimA type IV harboring strain.

### Rabbit Anti-FimI Ig Colocalization with *P. gingivalis* Inside the Cells

**Fig. 1:**
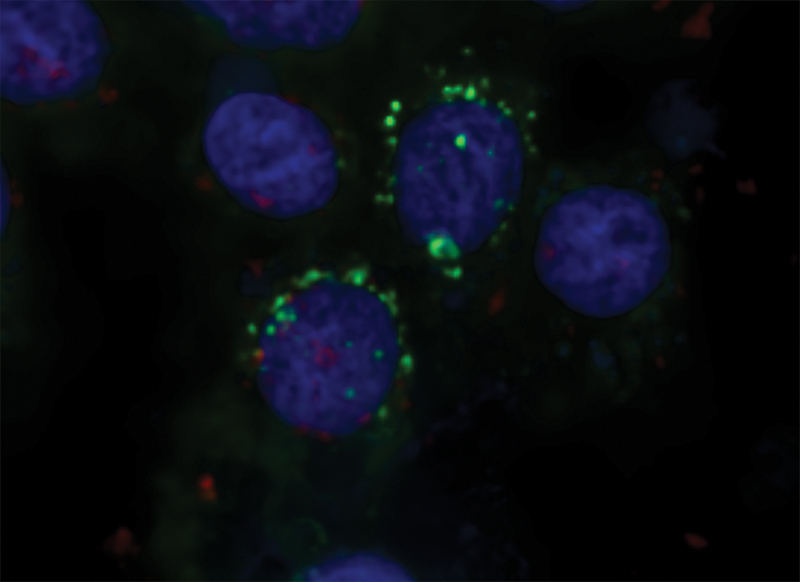
Confocal image of cells containing *Porphyromonas gingivalis* and anti-FimI immunoglobulin (Ig). Rabbit anti-FimI Ig-Cy3 was internalized into H357 cells that were infected with *P. gingivalis* -PKH67 by electroporation. One day after electroporation, cells were fixed and stained with DAPI, and visualized with confocal microscopy.

**Fig. 2:**
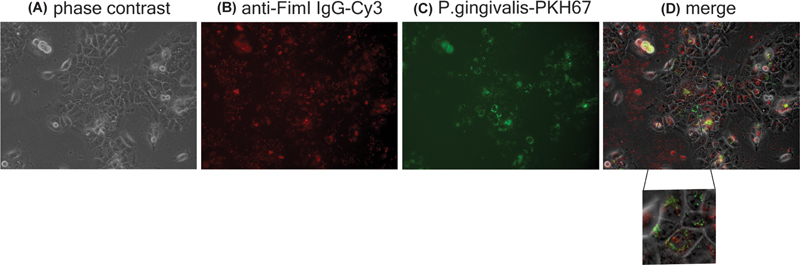
Localization of *Porphyromonas gingivalis* and anti-FimI immunoglobulin (Ig). Rabbit anti-FimI Ig-Cy3 was internalized into H357 cells that were infected with *P. gingivalis* -PKH67 by electroporation. Two days after electroporation, cells were imaged by fluorescence microscopy to visualize the cells in phase contrast ( **A** ), for Cy3 signal ( **B** ), and PKH67 signal ( **C** ) before merging ( **D** ).

To localize *P. gingivalis* and anti-FimI Ig inside the cells, H357 cells were infected with *P. gingivalis* labeling with PKH67 dye, and then anti-FimI Ig labeling with Cy3 was internalized into the infected cells by electroporation. One day after electroporation, cells were fixed and stained with DAPI, and visualized with confocal microscopy. In addition, 2 days after electroporation, cells were visualized with fluorescence microscope. Both *P. gingivalis* and the Ig were found colocalized near the nucleus ( Figs. 1 and 2 ). The same result was observed on day 3 but the fluorescence signal was weaker. On day 4, it was hard to observe fluorescence signal inside the cells as the signal was weak and the boundary of the cells was hard to locate after cell number was increased by cell proliferation.

### Rabbit Anti-FimI Ig Entered Cells by Coincubation

**Fig. 3:**
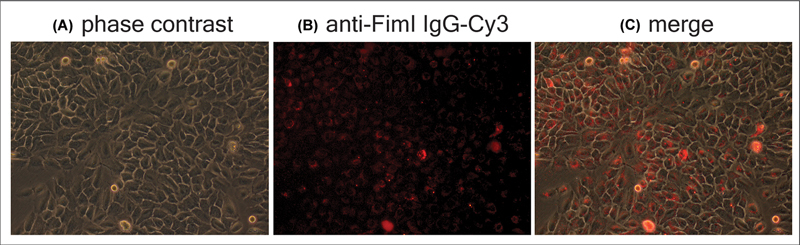
Localization of anti-FimI immunoglobulin (Ig) after coincubation with H357 cells. H357 cells that were infected with *Porphyromonas* *gingivalis* -PKH67 were cultured in the medium containing rabbit anti-FimI Ig-Cy3 for 16 hours. One day after the medium containing Ig was replaced with normal medium, cells were imaged by fluorescence microscopy to visualize the cells in phase contrast ( **A** ) and for Cy3 signal ( **B** ) before merging ( **C** ).

After culturing H357 cells in culture medium containing rabbit anti-FimI Ig for 16 hours, cells were observed with fluorescence microscope 1 day after the medium containing Ig was replaced with normal medium. Similar to that observed in the previous section, the Ig was found near the nucleus ( Fig. 3 ).

### Proportion of *P. gingivalis* Infected Cells after Anti-FimI Ig Internalization

Rabbit anti-FimI Ig was internalized into H357 cells containing *P. gingivalis* -PKH67 by electroporation. Percentage of the infected cells was observed using flow cytometry ( Table 1 ). The observation was done up to 6 days after electroporation since the level of fluorescence was low at day 6. Comparing with the control group with no electroporation, the electroporated cells group showed lower level of infected cells. At day 2 and 3, the percentage of infected cells were similar between the mock electroporation control group and the anti-FimI Ig electroporation group. At day 4, the percentage of infected cells in the anti-FimI Ig electroporation group seemed to be lower than that of the electroporation control group but the difference was not statistically significant.

**Table 1:** Percentage of *Porphyromonas gingivali* *s* infected cells after anti-FimI Ig internalization

	Percentage of H357 cells containing *P. gingivalis*		
	Control	Electroporation control	Electroporation with anti-FimI Ig
Day 2	22.25 ± 1.34	19.10 ± 0.92	18.90 ± 0.14
Day 3	21.45 ± 0.07	16.15 ± 0.49	16.35 ± 0.07
Day 4	20.5 ± 0.00	13.43 ± 0.28	12.83 ± 0.25
Day 6	12.67 ± 0.42	9.27 ± 0.31	9.03 ± 0.32

Abbreviation: Ig, immunoglobulin.

## Discussion

After H357 cells were infected with *P. gingivalis* , *P. gingivalis* cells were found near the nucleus, the location was speculated to be endoplasmic reticulum (ER). This was also found by another work, which showed that intracellular *P. gingivalis* localized to ER-perinuclear regions. 21 After electroporation or coincubation with rabbit anti-FimI Ig, the Ig was also found near the nucleus. The colocalization of the Ig and *P. gingivalis* makes a chance that the antibodies can bind to and inhibit the bacterial proliferation and survival.

The effect of the antibody to inhibition and killing of *P. gingivalis* is not clear in this study. Four days after the internalization of anti-FimI Ig into the infected cells, the infected cells seemed to be lower. However, the difference was not statistically significant. It might need to be investigated for a longer period of time, and the other technique of *P. gingivalis* fluorescence labeling will be required since the signal of PKH67 faded at day 6.

Rabbit anti-FimI Ig could enter H357 cells by only coincubation. Internalization of the antibody by this method might occur through the function of Ig receptor on H357 cell surface. However, there was no report of Ig receptor on H357 cells or normal oral epithelial cells. There are evidences showing that FcRn is expressed in normal human epidermal keratinocytes 22 and intestinal epithelial cells. 23 Fc gamma receptors also express in human nasal epithelial cells 24 and carcinoma cells. 25 It was shown that rabbit IgG could bind to human Fc gamma receptor. 26 If H357 cells have Ig Fc receptor on cell surface, anti-FimI Ig internalization after coincubation might be modified by this receptor.

## Conclusion

Rabbit anti- *P. gingivalis* FimI polyclonal antibody and *P. gingivalis* were colocalized near the nucleus. And the antibody was able to enter H357 cells by the coincubation method.
